# A genome-wide association study identifies a genomic region for the polycerate phenotype in sheep (*Ovis aries*)

**DOI:** 10.1038/srep21111

**Published:** 2016-02-17

**Authors:** Xue Ren, Guang-Li Yang, Wei-Feng Peng, Yong-Xin Zhao, Min Zhang, Ze-Hui Chen, Fu-An Wu, Juha Kantanen, Min Shen, Meng-Hua Li

**Affiliations:** 1 CAS Key Laboratory of Animal Ecology and Conservation Biology, Institute of Zoology, Chinese Academy of Sciences (CAS), Beijing 100101, China; 2 University of Chinese Academy of Sciences (UCAS), Beijing 100049, China; 3 Department of Life Sciences, Shangqiu Normal University, Shangqiu 476000, China; 4 Bureau of Animal Husbandry and Veterinary Medicine, Sishui County, Jining 273200, China; 5 Green Technology, Natural Resources Institute Finland (Luke), Jokioinen 31600, Finland; 6 Department of Biology, University of Eastern Finland, Kuopio 70211, Finland; 7 Institute of Animal Husbandry and Veterinary Medicine, Xinjiang Academy of Agricultural and Reclamation Sciences, Shihezi 832000, China; 8 Key Laboratory of Sheep Breeding and Development Technology of Xinjiang Production and Construction Crops (XPCC), Shihezi 832000, China

## Abstract

Horns are a cranial appendage found exclusively in Bovidae, and play important roles in accessing resources and mates. In sheep (*Ovies aries*), horns vary from polled to six-horned, and human have been selecting polled animals in farming and breeding. Here, we conducted a genome-wide association study on 24 two-horned versus 22 four-horned phenotypes in a native Chinese breed of Sishui Fur sheep. Together with linkage disequilibrium (LD) analyses and haplotype-based association tests, we identified a genomic region comprising 132.0–133.1 Mb on chromosome 2 that contained the top 10 SNPs (including 4 significant SNPs) and 5 most significant haplotypes associated with the polycerate phenotype. In humans and mice, this genomic region contains the *HOXD* gene cluster and adjacent functional genes *EVX2* and *KIAA1715*, which have a close association with the formation of limbs and genital buds. Our results provide new insights into the genetic basis underlying variable numbers of horns and represent a new resource for use in sheep genetics and breeding.

In mammals, most members of the infraorder Pecora, such as deer, antelope, cattle, goat, and sheep (*Ovies aries*), have cranial appendages. These appendages have four extant forms: antlers, horns, pronghorns and ossicones^1^. Horns, being exclusive to bovids, consist of a bony horn core covered by a scabbard-like keratinous sheath that is never shed^1^,^2^. Horns serve as a form of sexual weaponry for most domestic and wild animals; thus, larger horns have an advantage in strong intra-sexual competition^3^,^4^. However, horns may be disadvantages in livestock farming and breeding. For example, horned males may attack other animals or people and can get their heads stuck in fences and feeders. Therefore, hornless (or polled) individuals are preferred for animal farming and breeding.

A number of earlier studies have identified candidate genes for various traits in sheep, including coat colour, horn, meat production, growth and fecundity etc., using a genome-wide association approach^5^,^6^,^7^,^8^. In sheep, a majority of rams have strong and normal horns, while ewes can have smaller horns, scurs (deformed vestigial horns) or no horns (polled). Previous studies have primarily focused on polled versus horned phenotypes or on horn type, size and length in sheep and their wild relatives (e.g., *Ovis canadensis*)^3^,^4^,^8^,^9^,^10^. *Horns*, a single autosomal locus mapped to a 7.4 cM region on chromosome 10 (OAR10) in Soay sheep, has been demonstrated to control horn-type polymorphism and exerts a comparatively large effect on horn size^3^,^8^. Fine mapping studies identified relaxin-like receptor 2 (*RXFP2*), which contributes to main sex characters in humans and mice, as the major candidate gene for *Horns* in Soay sheep^3^,^4^. Quite recently, a 1.8-kb insertion in the 3′-UTR of *RXFP2* was identified to be associated with polledness in sheep^9^. Also, whole-genome resequencing of a wild sheep *Ovis canadensis* revealed that multiple populations shared a common selective sweep at *RXFP2*^10^.

However, the genetic basis of a different aspect of horn phenotype, the polycerate type (multiple horns), has not been examined. Globally, there are only a small number of polycerate native sheep breeds, including Jacob, Manx Loaghtan, Hebridean, Navajo-Churro, Icelandic sheep, Because nowadays there are no four-horned Finnish Landrace sheep. Altay sheep and Chinese Sishui Fur sheep (see Fig. 1). Horn phenotypes for the breeds range from polled to as many as six horns. Historically, four-horned sheep were widely distributed in both Europe and Asia^11^,^12^; however, they have become rare due to long-term artificial selection. Most of the four-horned sheep are rams, and four horns are rarely observed in ewes. The four-hornedness trait is dominant over two-hornedness, but recessive to polledness^12^. Sishui Fur sheep is a native sheep breed in eastern China, originating from ancient Mongolian sheep. They are fat-tailed and mainly distributed in pastoral and agricultural areas in Sishui County of Shandong Province. They can produce high-quality meat and carpet wool. Also, they have sound body conformation, strong walking ability and excellent adaptation to local ecological environments^13^. Rams of the breed typically have 0–6 horns, and the occurrence of 2 and 4 horns is most common (see Fig. 1). Ewes are always polled. However, the underlying mechanisms for the polycerate phenotype in rams of this and other sheep breeds have remained elusive.

Here, we carried out a genome-wide association (GWA) study and haplotype-based association tests to map a strong candidate genomic region for the polycerate phenotype in Sishui Fur sheep. Our results will help to elucidate the molecular mechanism underlying the trait and can be applied in molecular marker-assisted breeding programs.

## Methods

The methods were carried out in accordance with the approved guidelines of the Good Experimental Practices adopted by the Institute of Zoology, Chinese Academy of Sciences. All experimental procedures and animal collections were conducted under a permit (No. IOZ13015) approved by the Committee for Animal Experiments of the Institute of Zoology, Chinese Academy of Sciences, China.

### Sample collection and DNA extraction

Ear tissue samples were collected from a total of 60 rams of Sishui Fur sheep. Among them, 26 individuals with four horns were assigned as the case group, and the other 34 two-horned sheep were considered as the control group. All the samples were collected from stocks maintained on different farms in Sishui County, Shandong Province, China. Using both pedigree records and the farmers’ knowledge, particular efforts were made to ensure that all animals were typical of the breed and as unrelated as possible.

Genomic DNA was extracted from the ear tissues using a standard phenol/chloroform method^14^, and the DNA concentration was measured on a spectrophotometer (Nanodrop 2000, Wilmington, DE, USA). The extracted DNA was diluted to 100 ng/μl for SNP BeadChip genotyping.

### SNP genotyping and quality control

All sample DNA met the concentrations recommended for the Illumina Ovine Infinium HD SNP BeadChip genotyping according to the manufacturer’s protocol. Details on creation of the ovine BeadChip (685,734 SNPs) and genotyping procedures were described in Anderson *et al.*^15^. Genotypes for a total of 606,006 scorable SNPs, which passed the manufacturer’s quality control, were available for further analysis.

Stringent quality control parameters were applied for both the samples and SNPs to ensure reliability of results. We implemented the quality control measures using the software PLINK v1.07^16^. SNPs or individuals who met any of the following criteria were removed: (1) no chromosomal or physical location; (2) minor allele frequency (MAF) <0.05; (3) individuals call rate <0.9; (4) missing genotype frequency for SNP >0.05; (5) Fisher’s exact test^17^ *P*-value for Hardy-Weinberg equilibrium (HWE) <0.001. We further estimated pairwise relatedness using KING^18^,^19^, and closely related animals (e.g., full-sibs) were excluded from further analyses. After filtering, a total of 491,507 SNPs and 50 individuals (two-horned: 28 animals; four-horned: 22 animals) were left for the within-breed population stratification analysis.

### Population stratification and genome-wide association (GWA) analysis

Genetic differentiation between the case and control groups was assessed by Weir & Cockerham’s *F*_ST_ method^20^ using the program GENEPOP v4.2^21^, and multidimensional scaling (MDS) analysis using the program PLINK v1.07. The MDS analysis were implemented on a set of 15,911 independent SNPs pruned using the option of “indep-pairwise 50 5 0.05” in PLINK. This function calculates pairwise linkage disequilibrium (LD) in a 50-SNPs-window, shifts at a pace of 5 SNPs and excludes one of a pair of SNPs if the LD estimate *r*^2^ > 0.05. Results of the MDS analysis were plotted in the GenABEL package^22^ for R v3.2.2 (http://www.r-project.org). We further removed the 4 separated individuals from the control group in the following analyses (see Results). Our final working data set contained 46 individuals (24 two-horned; 22 four-horned).

Because the phenotype (2 or 4 horns) is binary, we performed a genome-wide association (GWA) study using the case-control model in the GenABEL package for R v3.2.2. Given the 15,911 pruned independent SNPs, the statistical threshold of genome-wide significance after Bonferroni correction was 3.14 × 10^−6^ (0.05/15,911) at the empirical level of 0.05^3^,^23^. To account for the GWA analysis confounded by population stratification, the genome-wide association *P*-values were corrected using the principal components (PCs)^22^,^23^. We used the top five principle components from the MDS analyses as covariates to adjust the systematic biases (see Results), which was also indicated by the Q-Q plot (quantile-quantile plot). We further conducted a resampling test (1,000 times) without replacement to determine the false positive rate (FPR) by chance using the function of “qtscore” in GenABEL. The command is detailed as follows:

qtscore(sample(phdata(data1)$dm2, replace = F), data1, trait.type = “binomial”), where “data1” is the gwaa.data object and “dm2” represents phenotype.

### Linkage disequilibrium (LD) and haplotype-based association analyses

The gametic LD measure of *r*^2^ among SNPs within the candidate genomic region (132–133.1Mb on OAR2, see Results) was calculated and visualised using the program Haploview v4.2^24^. LD blocks were defined based on the four-gamete rule algorithm^25^. Pairwise tests of LD for the most significant SNP rs420183358 with its flanking SNPs within approximately 1Mb upstream and downstream were obtained using PLINK v1.07. A regional association plot was generated using the R v3.2.2.

Further, we applied a haplotype-based association test to identify haplotypes significantly associated with the polycerate phenotype across OAR2. We first estimated the haplotypes and their frequencies in the LD blocks using the maximum likelihood (ML) method and expectation-maximization (EM) algorithm. Then, we performed the chi-square test for haplotype-phenotype association^26^, and statistical significance was also determined. We considered raw *P*-values (*P*_raw_) <1 × 10^−7^ as significant and *P*-values <0.05 after 100,000 permutations as genome-wide significant (i.e. *P*_genome_ <0.05). All the analyses were carried out using Haploview.

### Gene annotation

Genes within the candidate genomic region were determined using the *Ovis aries* assembly Oar_v4.0 (http://www.ncbi.nlm.nih.gov/genome/?term=ovis+aries/). However, given the lagging research of the sheep genome, the gene annotation in sheep is incomplete. We obtained additional functional information of orthologous genes in other species (e.g. human, mouse, chimpanzee, chicken and bovine) from UniProt (http://www.uniprot.org/) and published research regarding the candidate genes identified in sheep.

## Results

### Population stratification and significant SNPs by GWA analysis

**Figure 2:**
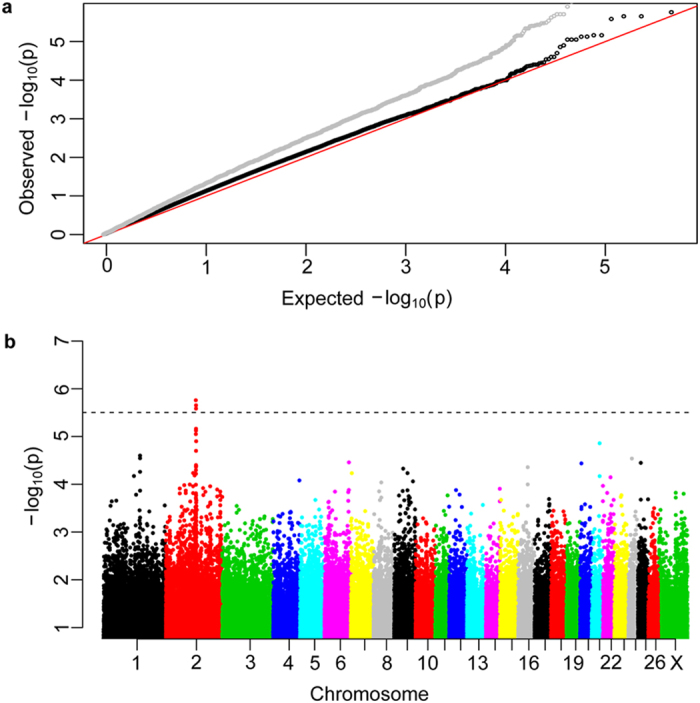
Q-Q plot and manhattan plot of genome-wide association (GWA) test. The “p” in the labels represents *P*-values of GWA analysis. **(a)** Q-Q plot: the grey and black dots denote association statistics before and after correction for population stratification, respectively; **(b)** Manhattan plot: the 5% genome-wide significant threshold value (*P* = 3.14  × 10^−6^) is presented by a black dashed line.

We obtained a *F*_ST_ value of 0.011 for the genetic differentiation between the case and control groups. Also, the MDS plot (see Supplementary Fig. S1 online) indicated within-population stratification, which may bias the association test. In particular, 4 two-horned animals of the control group showed apparent genetic differentiation from the rest animals (see Supplementary Fig. S1 online) and, thus, were removed from the GWA analysis. The raw genomic inflation factor lambda was 1.352 in the initial GWA analysis and turned to be 1.103 after applying the first five PCs in the population stratification correction (Fig. 2a).

All the top ten associated SNPs were identified as being located on OAR2 (Fig. 2b, Table 1) and extremely low FPR values (FPR <0.001) were observed for the SNPs after the bootstrapping test. We also detected statistically significant differences in allele frequencies of the ten SNPs between the case and control groups (paired *t*-test; *P* = 3.366 × 10^−7^, *t* = 13.128, d.f. = 9). Of the top ten SNPs, four (rs420183358, rs422753866, rs428812278 and rs428938943) showed significant associations with the polycerate phenotype at the genome-wide threshold of *P *< 0.05 (Table 1). SNP rs420183358 showed the most significant association (*P* = 1.74 × 10^−6^).

### LD and haplotype-based association test

**Figure 3:**
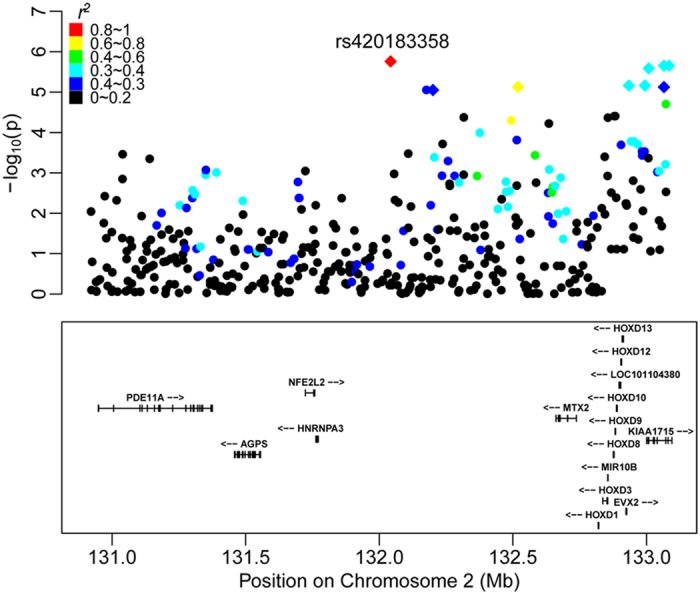
Plot of regional association results for loci surrounding the most significant SNP rs420183358 (red rhombus). Different colours represent the *r*^2^ values of pair-wise LD estimates. Functional genes in this region are plotted in the box. The rhombus represents the top ten SNPs of GWA analysis. The yellow dot represents rs403275219.

Pairwise tests of LD for the most significant SNP rs420183358 with neighbouring SNPs in its upstream and downstream regions indicated relatively strong LD (*r*^2^ > 0.6) with two SNPs (rs403275219 and rs416536940) (Fig. 3). A relatively high level of LD was observed across the pairwise SNPs in the candidate genomic region (Fig. 3). However, the two linked SNPs (rs403275219 and rs416536940) were distributed in a different haplotype block from that which contained the most significant SNP rs420183358.

Four-gamete rule algorithm identified a total of 1,552 LD blocks containing 44,373 haplotypes on OAR2. The chi-square test revealed that after 100,000 permutations 20 haplotypes were significant (*P *< 0.05) at the genome-wide threshold on the chromosome (Table 2). The most significant five haplotypes were distributed in 5 adjacent and strongly linked blocks (Table 2, Supplementary Fig. S2 online). Of the 5 most significant haplotypes, the top three haplotype of blocks 6085 (AGAG, *P *< 1 × 10^−5^), 6086 (AGGGA, *P *< 1 × 10^−5^) and 6084 (GAGCA, *P *< 1 × 10^−5^) had the same frequencies of 0.727 in the case groups versus 0.104, 0.104 and 0.125 in the control groups, respectively (Table 2). The results indicated that the region 132.9–133.1 Mb most probably harbours the causative mutations accounting for the polycerate phenotype.

### Gene annotation

Based on the lists of significant SNPs identified by GWA study and the haplotype-based association test, the 132.0–133.1Mb region on OAR2 was identified as a strong candidate genomic region for the polycerate phenotype. Thirteen genes have been annotated within this genomic region (see Supplementary Table S1 online). Metaxin 2 (*MXT2*) is a protein that is located on the cytosolic face of the mitochondrial outer membrane and works with metaxin 1 to import mitochondrial preproteins into mammalian mitochondria^27^. The *HOXD* gene cluster belongs to the homeobox family of genes, which encode a highly conserved family of transcription factors that specify differences in morphogenesis in all multicellular organisms^28^,^29^. Earlier studies in humans, mice and chicks have concluded that *5*′*HOXD* genes are closely associated with the development of limbs and genitalia^30^,^31^,^32^,^33^,^34^. *EVX2* is adjacent to *HOXD13*, i.e., 8 kb upstream, and forms the *EVX2*-*HOXD13* intergenic region, which behaves as a spatio-temporal boundary element^35^. *KIAA1715* is an orthologous gene to *LUNPARK* (*LNP*) in the human and mouse genomes, and it plays an important role in the development of digits and the central nervous system, along with *EVX2* and the *HOXD* cluster^30^. The most significant SNP rs420183358 identified here lies downstream of the *HOXD* genes, and the other three significant SNPs (rs422753866, rs428812278 and rs428938943) lie within the gene *KIAA1715*.

## Discussion

Understanding the genetic architecture of horn phenotypic variation in wild and domestic vertebrates is one of the fundamental goals in evolutionary genetics. In this study, we identified a genomic region for the polycerate phenotype using a GWA study approach.

The most significant SNP (rs420183358) is not located in any annotated gene, but downstream of the *HOXD* gene cluster. Thus, rs420183358 may be the causal mutation, which has a regulatory function. Alternatively, it is also possible that the causal mutation is located inside the *HOXD* gene cluster and is closely linked to the target SNP rs420183358. The candidate genomic region (132–133.1 Mb) revealed in this investigation is in accordance with an ongoing investigation on the genetic cause for the polycerate trait in Navajo-Churro and Jacob sheep, where a genomic region of 132Mb on OAR2 was identified as being associated with this trait^36^. This region is well-known for the *HOXD* gene cluster, which comprises nine genes (*HOXD1*, *HOXD3*, *HOXD4* and *HOXD8-HOXD13*) in order from 3′ to 5′. *HOXD* genes are the primary determinants of the anterior-posterior body axis in all bilaterians, including the trunk or appendicular axis^37^,^38^. *HOXD11* is expressed along the primary body axis, and *HOXD11* mutant mice (or mice with a deletion of *HOXD13* to *HOXD11*) exhibit a supernumerary lumbar vertebra^39^,^40^. *HOXD13* and *HOXA13* are most strongly expressed at the distal ends of limbs and genital buds, and the absence of the two genes could affect the development of hands and feet^31^,^32^,^34^. Synpolydactyly, a limb malformation, is caused by *HOXD13* mutations^41^, and later research suggested that removing *HOXD9*-*HOXD13* and *EVX2* also led to synpolydactyly^35^. *EVX2* is located 8kb upstream of *HOXD13* and responds to the digit enhancer in a similar way as the *HOXD* genes^35^,^42^.

The significantly associated haplotypes were distributed in several LD blocks (see Supplementary Fig. S2 online). The three most significant haplotypes (in blocks 6084–6086) containing three significant SNPs (rs422753866, rs428812278 and rs428938943) were located within the gene *KIAA1715*, which has the functions associated with the formation of limb digits and genital buds^30^,^41^,^43^. The *HOXD* gene cluster and genes *EVX2* and *KIAA1715* are located in the candidate genomic region in a successive order (Fig. 3). All these genes have analogous functions in limb and digit development, and the number of digits and lumbar vertebras are controlled by this region^30^. Also, previous work localized a “digit enhancer” upstream from the *HOXD* cluster^39^. Subsequent studies demonstrated that a global control region (GCR) defined a chromosomal regulatory landscape containing the *HOXD* gene cluster, with *LNP*, *EVX2* and *HOXD*-specific patterns^30^. Therefore, the GCR could also regulate the polycerate phenotype.

Our approaches for detecting the candidate genomic region underlying the polycerate phenotype were efficient, although the relatively small sample size was one possible potential limitation of this study. Future investigations with a larger sample size in other sheep breeds and deep-sequencing of the candidate genomic region are necessary to confirm and extend our findings. In conclusion, our results provided strong evidence that the genomic region 132.0–133.1 Mb on chromosome 2 is associated with the polycerate phenotype.

## Additional Information

**How to cite this article**: Ren, X. *et al.* A genome-wide association study identifies a genomic region for the polycerate phenotype in sheep (*Ovis aries*). *Sci. Rep.* **6**, 21111; doi: 10.1038/srep21111 (2016).

## Supplementary Material

Supplementary Information

## Figures and Tables

**Figure 1:**
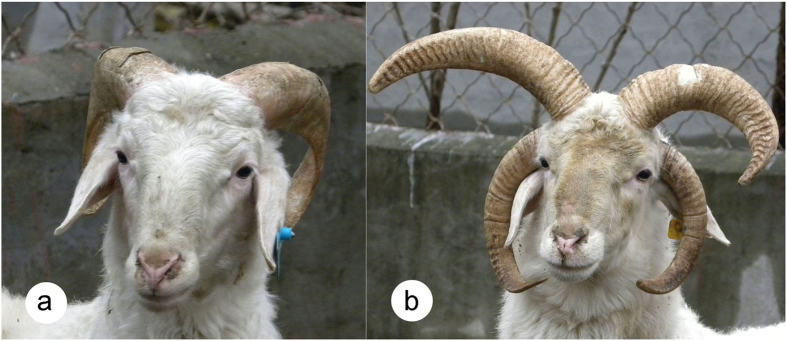
Sishui Fur sheep with the **(a)** two-horned and **(b)** four-horned phenotypes.

**Table 1:** The top ten SNPs associated with the polycerate phenotype.

Chr.	SNP	Position	Allele	MAF	Frequency		*P*-value	FPR
					Case	Control		
2	rs420183358	132042535	A:G	0.222	0.405	0.062	1.74E-06	<0.001
2	rs422753866	133065296	A:G	0.402	0.727	0.104	2.221E-06	<0.001
2	rs428812278	133084414	G:A	0.402	0.727	0.104	2.221E-06	<0.001
2	rs428938943	133008470	G:A	0.413	0.727	0.125	2.601E-06	<0.001
2	rs429526398	132934057	G:A	0.413	0.705	0.146	6.876E-06	<0.001
2	rs419153948	132994229	G:A	0.413	0.705	0.146	6.876E-06	<0.001
2	rs416536940	132519432	A:G	0.217	0.386	0.062	7.482E-06	<0.001
2	rs403336550	133065371	G:A	0.446	0.75	0.167	7.518E-06	<0.001
2	rs418381175	132200980	G:A	0.391	0.818	0.417	8.912E-06	<0.001
2	rs408254270	132201113	G:A	0.391	0.818	0.417	8.912E-06	<0.001

Chr. is the abbreviation format of chromosome. MAF represents minor allele frequency. *P*-value represents the corrected significance of GWA after principle component adjustment. FPR means false positive rate.

**Table 2:** The top ten significant haplotypes with the polycerate phenotype.

LD Block	Haplotype	Position		Frequency		*P*-value	*P*_100,000_
		first	last	Case	Control		
6085	AGAG	133040292	133065679	0.727	0.104	1.14E-09	<0.00001
6086	AGGGA	133072339	133084414	0.727	0.104	1.14E-09	<0.00001
6084	GAGCA	133008423	133017771	0.727	0.125	4.61E-09	<0.00001
6081	ACCGA	132934057	132951006	0.705	0.146	5.43E-08	0.0005
6083	AAAG	132978078	132994332	0.705	0.146	5.43E-08	0.0005
11095	GA	240198021	240198140	0.136	0.646	6.42E-07	0.0096
6125	GGAG	133917957	133928107	0.455	0.021	7.38E-07	0.0105
6107	GGGG	133560053	133564962	0.841	0.333	8.60E-07	0.0120
11110	AGACC	240440866	240460254	0.592	0.106	8.82E-07	0.0120
6097	GCGGGG	133339051	133365628	0.523	0.062	9.72E-07	0.0128

*P*_100,000_ represents *P*-value with 100,000 permutations in the association tests; LD denotes linkage disequilibrium.
