# Erratum: A genome-wide association study identifies a genomic region for the polycerate phenotype in sheep (*Ovis aries*)

**DOI:** 10.1038/srep25322

**Published:** 2016-05-20

**Authors:** Xue Ren, Guang-Li Yang, Wei-Feng Peng, Yong-Xin Zhao, Min Zhang, Ze-Hui Chen, Fu-An Wu, Juha Kantanen, Min Shen, Meng-Hua Li

Scientific Reports 6: Article number: 21111; 10.1038/srep21111 published online: 02172016; updated: 05202016.

In this Article, there is an error in the Introduction section.

“Globally, there are only a small number of polycerate native sheep breeds, including Jacob, Manx Loaghtan, Hebridean, Navajo-Churro, Icelandic sheep, Because nowadays there are no four-horned Finnish Landrace sheep. Altay sheep and Chinese Sishui Fur sheep (see Fig. 1)”.

should read:

“Globally, there are only a small number of polycerate native sheep breeds, including Jacob, Manx Loaghtan, Hebridean, Navajo-Churro, Icelandic sheep, Altay sheep and Chinese Sishui Fur sheep (see Fig. 1)”.

