# Supplementary material for: A genome-wide association study identifies a genomic region for the polycerate phenotype in sheep (Ovis aries)
Source: Sci Rep. 2016 Feb 17;6:21111. doi: 10.1038/srep21111 (PMC4756668; doi:10.1038/srep21111)
Supplement: Supplementary Information [file srep21111-s1.pdf]

Supplementary Materials

A genome-wide association study identifies a genomic region for the polycerate phenotype in sheep (*Ovis aries*)

Xue Ren[1,2*], Guang-Li Yang[1,3*], Wei-Feng Peng[1,2], Yong-Xin Zhao[1,2], Min Zhang[1], Ze-Hui Chen[1,2], Fu-An Wu[4], Juha Kantanen[5,6], Min Shen[7,8] & Meng-Hua Li[1]

[1]CAS Key Laboratory of Animal Ecology and Conservation Biology, Institute of Zoology, Chinese Academy of Sciences (CAS), Beijing 100101, China

[2]University of Chinese Academy of Sciences (UCAS), Beijing 100049, China

[3]Department of Life Sciences, Shangqiu Normal University, Shangqiu 476000, China

[4]Bureau of Animal Husbandry and Veterinary Medicine, Sishui County, Jining 273200, China

[5]Green Technology, Natural Resources Institute Finland (Luke), Jokioinen 31600, Finland

[6]Department of Biology, University of Eastern Finland, Kuopio 70211, Finland

[7]Institute of Animal Husbandry and Veterinary Medicine, Xinjiang Academy of Agricultural and Reclamation Sciences, Shihezi 832000, China

[8]Key Laboratory of Sheep Breeding and Development Technology of Xinjiang

Production and Construction Crops (XPCC), Shihezi 832000, China

*Equal contribution

Correspondence and requests for materials should be addressed to M.-H. L.

(menghua.li@ioz.ac.cn)

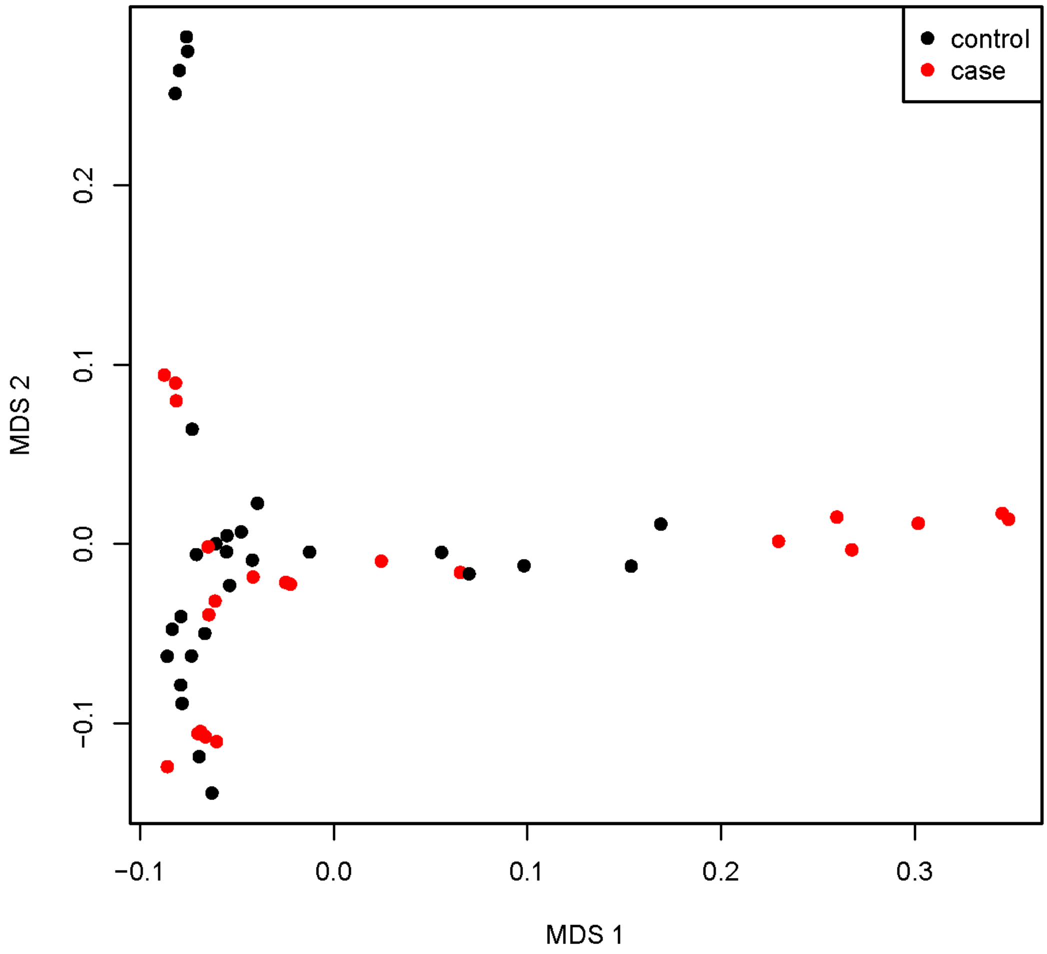

Supplementary Figure S1. Multidimensional scaling plot (MDS plot). The black dots represent animals in the control group, and the red dots indicate animals from the case group.

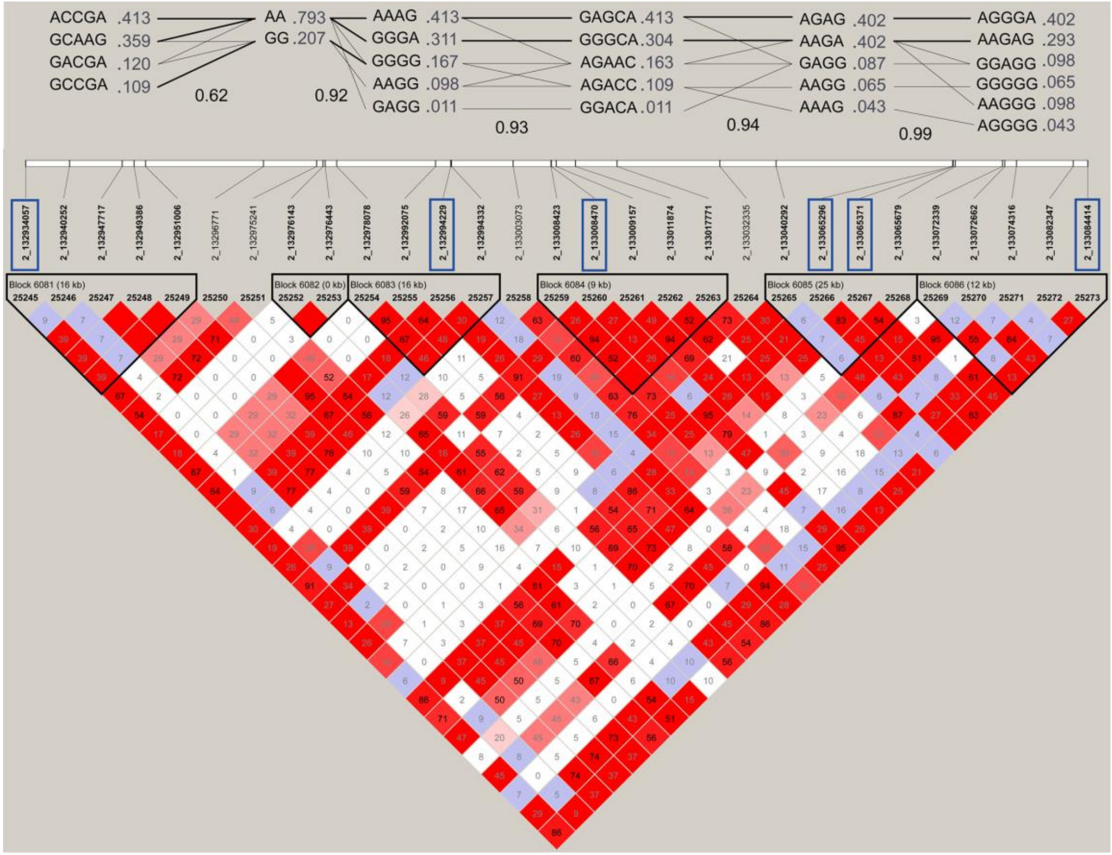

Supplementary Figure S2. The significantly associated haplotypes and LD blocks visualised using Haploview v4.2. The blocks were defined by four-gamete rule. r^2 values are presented in colourful squares. Haplotypes with population frequencies are upside of each block. In the crossing areas, multiallelic *D*' values indicate the level of recombination between the two blocks. SNPs (denoted by position on chromosome 2) in blue boxes from left to right are the significant SNPs (rs429526398, rs419153948, rs428938943, rs422753866, rs403336550 and rs428812278, see also Table 1) detected by genome-wide association (GWA) analysis.

Supplementary Table S1. Functional genes in the candidate genomic region (132.0 -133.1 Mb) on chromosome 2

**Gene**	**Start (bp)**	**Stop (bp)**	**Strand**	**Description**
LOC105609044	132190083	132197376	+	uncharacterized LOC105609044
LOC105609042	132210048	132363017	-	uncharacterized LOC105609042
LOC106990953	132345268	132350981	+	uncharacterized LOC106990953
LOC105609047	132434763	132443283	+	uncharacterized LOC105609047
MTX2	132672354	132748945	-	metaxin 2
HOXD1	132830789	132832890	-	homeobox D1
LOC105609052	132842815	132843657	+	uncharacterized LOC105609052
LOC105609054	132845690	132847753	-	uncharacterized LOC105609054
HOXD3	132847778	132866477	-	homeobox D3
MIR10B	132868982	132869080	-	microRNA mir-10b
LOC105610367	132876480	132884305	+	myosin heavy chain IB-like
HOXD8	132884968	132888875	-	homeobox D8
HOXD9	132894431	132899468	-	homeobox D9
HOXD10	132899503	132902661	-	homeobox D10
LOC101104380	132909273	132915738	-	homeobox protein Hox-D11
HOXD12	132916631	132919361	-	homeobox D12
HOXD13	132922784	132926088	-	homeobox D13
EVX2	132934176	132938463	+	even-skipped homeobox 2
LOC101107331	132987715	132988173	-	uncharacterized LOC101107331
KIAA1715	133011855	133110982	+	KIAA1715 orthology
